# Easy-to-Make Polymer Hydrogels by UV-Curing for the Cleaning of Acrylic Emulsion Paint Films

**DOI:** 10.3390/polym13132108

**Published:** 2021-06-26

**Authors:** Irene Cárdaba, Luca Porcarelli, Antonela Gallastegui, David Mecerreyes, Miren Itxaso Maguregui

**Affiliations:** 1 Painting Department, Faculty of Fine Arts, University of the Basque Country (UPV/EHU), Barrio Sarriena S/N, 48940 Leioa, Bizkaia, Spain; itxaso.maguregui@ehu.eus; 2 POLYMAT, Basque Center For Macromolecular Design and Engineering, University of the Basque Country UPV/EHU, Avenida Tolosa 72, Donostia, 20018 San Sebastián, Gipuzkoa, Spain; l.porcarelli@deakin.edu.au (L.P.); antonela.gallastegui@polymat.eu (A.G.); david.mecerreyes@ehu.es (D.M.)

**Keywords:** acrylic paint, UV-cured hydrogels, art conservation, cleaning

## Abstract

The cleaning of acrylic emulsion paint surfaces poses a great challenge in the conservation field, due to their high water sensitivity. In this article, we present easy-to-make polymer hydrogels, made by UV-photopolymerization, that show excellent cleaning properties. The formulation of hydrogels obtained by UV-curing and their performance as dry cleaners for acrylic paints was investigated. First, different hydrogel formulations based on functional acrylic monomers were used to formulate a series of UV cross-linked hydrogels by fast UV photopolymerization. Their effectiveness on surface dirt removal was investigated by SEM microscopy and colorimetry. The hydrogels showed excellent cleaning properties and controlled water release, and they still performed satisfactorily after several cleaning uses. The obtained UV-hydrogels were compared to the well-known agar gels, showing benefits in terms of reducing excess water. This article shows that easy-to-make UV-cured hydrogels are an efficient tool for the cleaning of surface dirt from water-sensitive paintings, overcoming the limits of traditional cleaning methods.

## 1. Research Aim

The aim of this work is to make the first approach to improving the cleaning methodology of water-sensitive paint surfaces by using fast, easy-to-make, and tunable UV-cured hydrogels. The main advantage of the proposed method is that UV-curing is a fast and available method [1,2,3,4] with high success in the coating industry and 3D printing, as well as other daily applications in health and esthetics. Besides the chemical compounds, it only needs a UV-lamp for obtaining the needed material in a rapid manner. Thus, avoiding complex laboratory processes while optimizing the preparation times is what makes these gels more affordable for the restorer than other types of hydrogels or cleaning materials.

In this work, we assessed the cleaning performance of several acrylic hydrogels. To do this, we investigated several formulations of UV-cured hydrogels based on commercially available functional (meth)acrylates, in order to find the most effective formulation for removing surface dirt while not harming the paint surface. The second part of the work compares the cleaning capability of UV hydrogels with the well-known agar gel. Agar is a commonly used and easy-to-make system in the conservation of art which has shown several drawbacks in terms of water retention capability [5]. This study shows the improvement in terms of water containment when using the UV-cured hydrogels presented here.

## 2. Introduction

The cleaning of water-sensitive paintings such as acrylic emulsion paint films is a very common practice in the conservation of contemporary art. The gradual accumulation of surface grime on paintings is a very common concern everywhere that art is present [6]. Even in a presumably benign museum environment, the slow accumulation of surface grime leads to the discernible visual alteration of a painted surface after approximately 50 years [6,7]. Therefore, we can assume that most of the artworks made out of acrylic paint, which was first introduced in 1953 by Rohm and Haas [8], are now prone to needing surface cleaning. In fact, many contemporary artists employed acrylic paints as their main media to create artworks [9]. Moreover, the vast production of contemporary painters has filled museum collections with these kinds of works [10]. However, this apparently simple conservative intervention is a delicate and challenging operation, due to, on the one hand, the complexity of the paint system itself [6], and on the other hand, its high water sensitivity [11,12,13,14,15,16].

Acrylic emulsion paints are made from water, acrylic monomers, stabilizers, thickeners, coalescing agents, pigments, and a wide range of additives that improve the paint’s properties [6,17]. The resulting film can become tacky at room temperature due to a low glass transition temperature (T*g*), which triggers the adhesion of airborne dust [11,17]. Moreover, static charges and hydrophilic additives accumulated in the surface, such as surfactants, can attract and embed environmental soil [11,18,19]. Research carried out by a number of conservation scientists [13,14,16,17,18,19,20,21,22,23,24,25,26,27], identified the factors that influence the sensitivity of acrylic paint films to organic solvents and water-based cleaning systems [6,11]: additive migration, swelling of the paint film, and re-transport of solubilized matter through the porous matrix of the artifacts during the cleaning procedure [12,13,14,15] are, among other factors, only a few of the obstacles ahead.

According to some authors, the swelling of the paint corresponds to the sensitivity of the thickeners that are part of the composition [6]; others stated that water sensitivity is related to the quantity of non-ionic surfactants that are commonly present in acrylic paint surfaces [18,22].

Several authors agree that the modification of the pH and the conductivity in a cleaning solution can help to control water absorption and material extraction [6,18,28,29,30,31,32,33,34]. In an alkaline or neutral environment, acrylic film hydrates and swells, softening and causing disruption [31]. Therefore, to minimize the swelling, the pH of the cleaning system must be kept as low as possible [6]. Furthermore, the ionic strength needs to be controlled. Both the cleaning solution and the paint layer have to be isotonic, in order to avoid the movement of ions between layers [6]. All this leads to large problems in finding accurate and safe cleaning systems, and the subsequent need to investigate new cleaning methods [20,32].

The controlled release of water needs to be considered as a tool to improve effectivity, especially when non-confined solvents and solutions are employed [15,16]. Therefore, the use of confined cleaning fluids within a gel matrix that releases them in a controlled way onto the surface of the work of art has been proposed [15,16]. In recent years, several methods have been tested to improve the control of cleaning fluids. Some researchers proposed thickeners, such as cellulose derivatives and polyacrylic-based formulations [33], to enhance the control of solvent diffusion by increasing the viscosity of the liquid [15,16,33,34]. However, the residue in thickened systems is still a challenge that has not been resolved [17,35,36]. Rigid gels, which can be classified as physical or chemical, according to their bonding nature [15,37], were proposed as residue-free confining systems. Polysaccharides such as gellan gum and agar are the most popular gelling agents employed to obtain the rigid physical gels used by conservators/restorers [15,38], due to their fast and easy preparation and handling. However, they retain high amounts of water, making control over the liquid release problematic [39,40,41].

As an alternative, xPVOH-borax gels [42,43], freeze-thaw PVA gels [44], and pHEMA/PVP [5,39] chemical hydrogels have emerged, proving to be more efficient, safe, retentive and tunable for the cleaning of water-sensitive artifacts.

However, the manufacture of these chemical gels usually requires complex chemical processes, with the exception of the borax gels, and are therefore less integrated (extended) into the daily work of the restorer.

This paper aims to be the first approach to introducing UV-cured hydrogels using commercially available acrylic monomers, in order to achieve a system with good cleaning properties and with the ease of being able to prepare them in your own workspace.

## 3. Materials and Methods

### 3.1. Materials

2-(Hydroxyethyl)methacrylate (HEMA) (assay 97%-) vinylpyrrolidone (VP), [2-(methacryloyloxy)ethyl]trimethylammonium chloride (METAC) 75 wt % in H_2_O, 2-acrylamido-2-methyl-1-propanesulfonic acid (AMPS) (99%), poly(ethylene glycol) dimethylmethacrylate (PEGDMA, MW 550 g mol^−1^), 2-hydroxy-4′-(2-hydroxyethoxy)-2-methylpropiophenone (IRGAcure 2956), glacial acetic acid (99.99%), and ammonium hydroxide solution (28% NH_3_ in H_2_O), were all supplied by Sigma Aldrich (St. Louis, MO, USA) and used as received for the synthesis of the UV-cured hydrogels. Agar-agar was provided by C.T.S. España. S.L. Acrylic paint mock-ups were prepared using brown PBr7 and titanium white PW6 paints from Liquitex^®^ (Cincinnati, OH, USA).

### 3.2. Preparation of Hydrogels by UV-Photopolymerization

**Figure 1:**
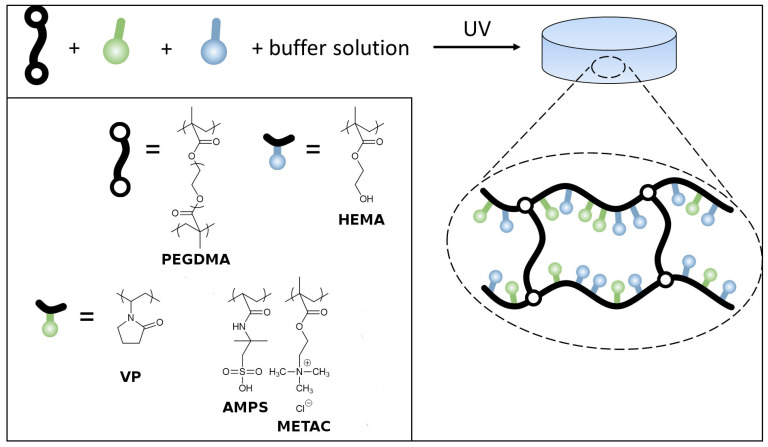
UV-cured hydrogels for the cleaning of water-sensitive paintings: reaction scheme and chemical structure of the monomers used and representation of a UV-cured hydrogel.

Chemically cross-linked UV-cured hydrogels were synthesized by photo-polymerization according to the reaction shown in Figure 1. In a typical reaction, for 4 g of hydrogel solution, 1.79 g of HEMA, 0.2 g of VP, 0.01 g of PEGDMA and 0.04 g of the photoinitiator IRGAcure 2956 were dissolved in 2 g of pH-adjusted water. All the components were stirred together except the photoinitiator, which was added just before the UV exposure. Different hydrogels were designed by varying the proportions of monomer/cross-linker ratio, photoinitiator percentages, and the pH of the adjusted water. Preliminary tests were performed to find the most adequate copolymer compositions and monomer/crosslinker ratio. The compositions of the hydrogels selected for the cleaning trials are presented in Table 1. Components were chosen for their affinity with water to create hydrogels. Different monomers were tested in order to check the cleaning capacity of their different functional groups. HEMA and VP were chosen to obtain a water-insoluble network, as they comprise two reactive groups capable of cross-linking the hydrogel acrylate backbone [5]. METAC and AMPS were used as monomers in order to improve cleaning efficiency while maintaining the good mechanical properties of the structure (stickiness and flexibility). PEGDMA was added as a crosslinker in order to ensure mechanical stability without stiffening the hydrogel [3]. Two water buffers of pH 5.0 and 6.5 were prepared for comparison. The highest and lowest pH values were selected according to the safety range proposed by Stavroudis in the modular cleaning program (MCP) recipes for the cleaning of acrylic paints [6,45]. Both buffers were adjusted in conductivity to 6.0 mS/cm², as advised elsewhere [17,45].

The solution was irradiated with UV light for 15 min for polymerization. A UV chamber from Boekel Scientific was used for hydrogel crosslinking, model 234100. Crystal vials were used as molds, obtaining 2.5-cm diameter and 1-cm thick gels.

### 3.3. Acrylic Paint Mock-Ups

Acrylic paint was applied on Mylar sheets as described elsewhere [17]. Two commercial paints (Liquitex white PW6 and brown PBr7) were selected to evaluate the cleaning efficiency of the method. Both colors were chosen in order to detect the variable results that may be obtained according to the different nature of the pigments. White samples were employed to test the cleaning efficiency, and brown paint was used to test the sensitivity toward the cleaning method. Some samples were kept as reference and others were artificially soiled according to published protocols [24], using a mixture of carbon black, iron oxide, silica, kaolin, gelatin powder, soluble starch, cement, olive oil, mineral oil and petroleum spirit, in the stated proportions [24]. Cleaning tests were conducted after three years [24] of natural aging. The excess loose soil was removed with a brush and the gel cleaning tests were performed on the embedded soiling layer. All mock-ups were stored in dark conditions and a dust-free environment.

### 3.4. FTIR

A Brueker ALPHA FTIR spectrometer was used to record IR spectra of the synthesized hydrogel. The spectra were collected using 32 scans with a resolution of 2 cm^−1^ in the ATR mode. An average spectrum was obtained out of several measurements taken from the surface of the hydrogels, in order to probe the complete curing of the hydrogels.

### 3.5. Colorimetry

Color measurements were carried out using a Konica-Minolta spectro-colorimeter, model CM700d. It was equipped with an integrating sphere, d/8° measurement geometry, and worked in the 400–700 nm spectral range with a 10 nm acquisition step. The light source and detector were, respectively, a pulsed xenon lamp with a UV cut filter and a silicon photodiode array. The instrument was provided with its own white reference (100% reflective) and a zero-calibration box (0% reference). Color measurements were acquired using D65 illuminant, a 10° supplementary standard observer, and excluded the specular component of light. The data reported were based on an average of three measurements and were calculated for the CIE L*a*b* 1976 color space. For each measurement, the spectro-colorimeter was positioned in exactly the same spot (Ø 8 mm) for each sample. Color differences, ∆(L*a*b*) and ∆E [46], were calculated on average values.

### 3.6. Scanning Electron Microscope

Scanning electron microscope (SEM) pictures were acquired using a ΣIGMA (Carl Zeiss Microscopy GmbH, München, Germany) scanning electron microscope with an acceleration potential of 5 kV. Acrylic paint film mock-ups were gold-metalized using an Agar Scientific auto sputter coater.

## 4. Results and Discussion

### 4.1. Characterization of UV-Cured Hydrogels

Eight different hydrogels were synthesized by UV-photopolymerization according to the reaction scheme shown in Figure 1. By this method, a ready-to-use hydrogel is directly obtained in less than 30 min. As shown in Table 1, each hydrogel was named according to the constituent monomers, followed by the pH value of the aqueous part.

**Figure 2:**
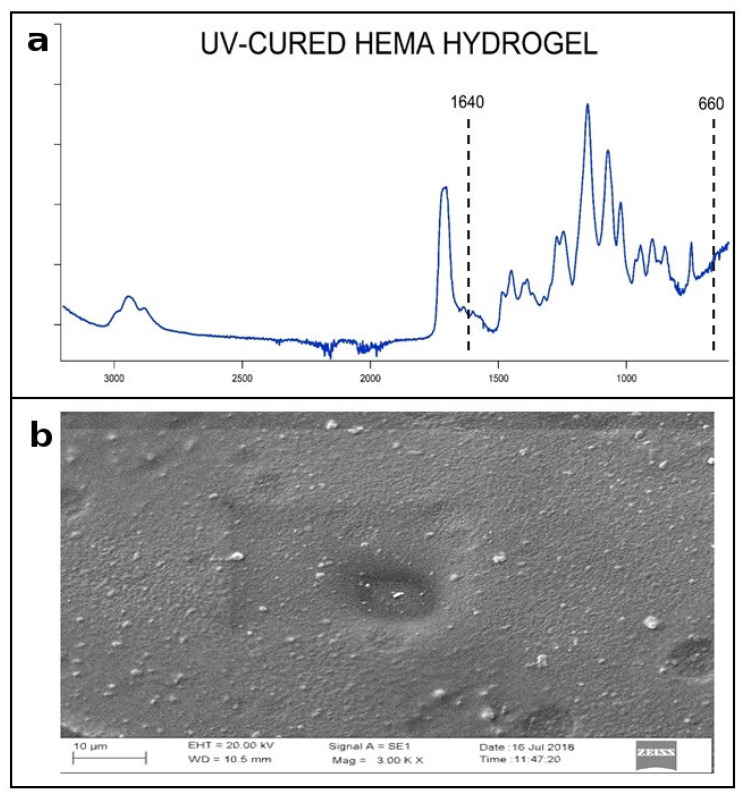
(**a**) FTIR-ATR spectra of HEMA_5. (**b**) SEM image of HEMA_5 gel surface.

The UV-curing degree of the hydrogels was characterized by using FTIR-ATR, as shown in Figure 2a, which depicts a typical FTIR-ATR spectrum of a hydrogel. The spectrum shows the distinctive stretch of the ester carbonyl group C=O at 1729 cm^−1^ and the distinctive CO ether stretches at 1096 cm^−1^ associated with the acrylic polymer network. Other bands, corresponding to the CH stretch at 2866 cm^−1^, CH2 bending stretch at 1450 cm^−1^, and CH3 bending stretch at 1350 cm^−1^, were also observed. Interestingly, quantitative monomer polymerization was demonstrated by the disappearance of the characteristic C=C stretch band of the acrylic groups in the fingerprint region at 1640 and 660 cm^−1^. All in all, the hydrogel formation through the effect of the radical photopolymerization process induced by the UV-curing is confirmed. Next, scanning electron microscopy (SEM) images were taken in order to characterize the surface morphological features of the UV-cured hydrogels. Figure 2b shows one typical image showing a homogeneous smooth surface of a representative HEMA-VP pH 5 hydrogel. All the hydrogels showed similar SEM images, and none of the samples showed relevant morphological features that could affect their cleaning performance.

Gravimetric measurements determined the water retention/release capability of the hydrogel. After gently drying the hydrogel with blotting paper, a fully swollen hydrogel was weighed and placed in a Petri dish over absorbent paper and covered with a mylar sheet to avoid water evaporation. After 30 min, water release was determined by the weight difference of the hydrogel before and after its application (Table 2). The measurements were repeated three times.

It was also necessary to assess the optical characteristics of the hydrogels according to the needs of the art conservators. Therefore, the selected hydrogels were those with the most adequate physical properties (transparency, stickiness, malleability, flexibility) for performing the cleaning. Transparent hydrogels were preferred, since they allow better control of soil removal from the work of art. HEMA_5 and HEMA_6.5 were too opaque, while HEMA_METAC5 and HEMA_METAC6.5 were more rigid and less sticky. On the contrary, both HEMA_VP and HEMA_AMPS gels are more malleable.

**Figure 3:**
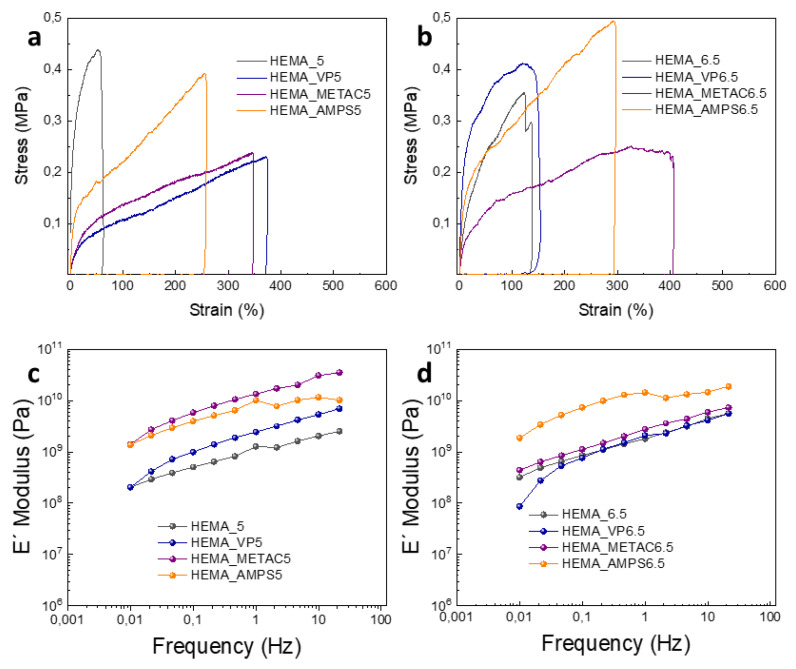
Tensile test (**a**,**b**) and dynamic mechanical analysis (DMA) (**c**,**d**), employing the hydrogels prepared with buffer pH 5 and buffer pH 6.5.

The mechanical properties of the hydrogels were analyzed by tensile tests and dynamic mechanical analysis (DMA) tests. In Figure 3a,b, the results of controlled tension versus elongation of the hydrogels prepared in buffers 5 and 6.5 can be observed. At pH 5, the hydrogels based on poly(HEMA) homopolymer show maximum elongation at a break of around 50%. The hydrogels based on the HEMA, including other comonomers such as VP, METAC and AMPS, show higher elongation at break values between 200% and 400% but are shorter. A similar trend is observed for the hydrogels prepared with a buffer of a pH of 6.5. In addition, DMA tests were carried out to study the behavior of these hydrogels against a frequency sweep (Figure 3c,d). The storage modulus (E′) of the hydrogels synthesized with both buffers showed values between 1 × 10^9^ and 1 × 10^10^ Pa at 1 Hz of frequency, high values that allow hydrogels to be considered as rigid materials. These results, together with the tensile tests, are indicative that these hydrogels are soft, elastic and ductile materials with sufficient rigidity to be used with little need for care.

### 4.2. Application and Maintenance of the Hydrogels

UV-cured hydrogels can be applied both dry (directly after synthesis) or loaded (after 24-h immersion in a water-based cleaning solution). If the gel is used water-loaded, it is recommended to blot the gel with absorbent paper before application in order to remove the excess liquid.

The swelling capability of the hydrogels and their water uptake depends on their chemical composition. After 24-h immersion in water, HEMA hydrogels showed 0% water uptake, HEMA-VP at 11%, HEMA-METAC at 43% and HEMA-AMPS up to 52%, respectively.

Gels can be manipulated using tweezers and the conservator’s own hands, always with clean gloves. For the application, place the gel on the surface that needs to be cleaned and apply a little pressure to enhance the adhesion between the gel and the surface. A plastic film can be placed over the gel to reduce the evaporation of water during the cleaning process.

Gels should be stored, whether dry or immersed in water, in closed containers. They can be kept in dark conditions at room temperature for several months after the synthesis. It is advisable to check gels after a long storage time, in order to verify that no alteration has occurred.

### 4.3. Cleaning Efficiency of UV-Cured Hydrogels

All the hydrogel formulations described in Table 1 were tested in order to assess the cleaning performance when applying the hydrogels on artificially soiled mock-ups. Cleaning tests were carried out by direct application of the UV-cured hydrogels onto the soiled paint layers for 20 s. Application time can be adjusted according to the specific needs of the surface. Good mechanical properties allow easy handling and it is worth noting that they can be directly applied to the surface of the painting. UV-cured hydrogels were used as prepared, without the need for any additional loading of buffered aqueous solutions, thus reducing the water content.

**Figure 4:**
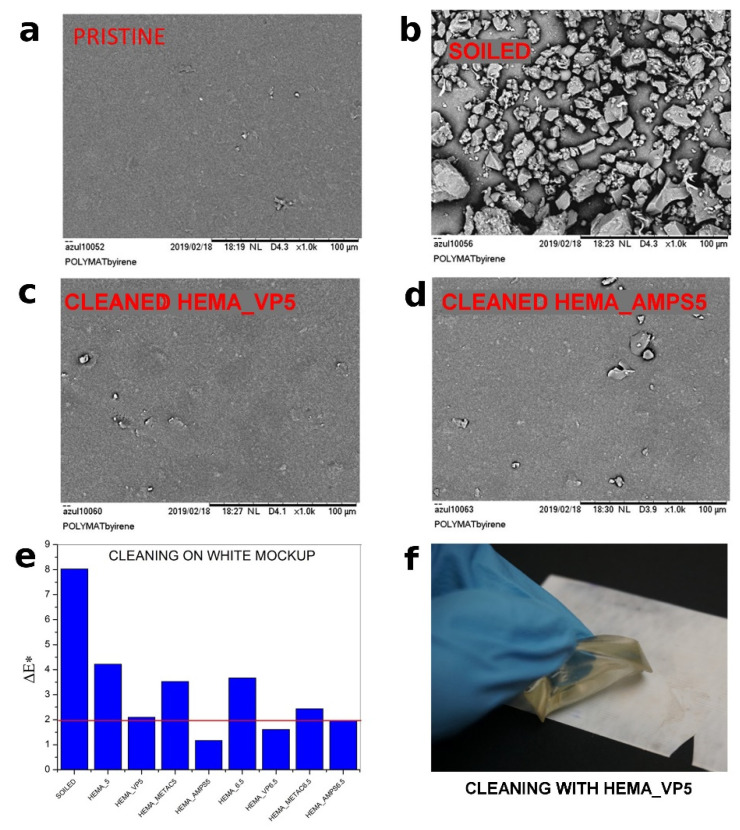
(**a**–**d**) SEM images before and after cleaning of white Liquitex mock-ups using HEMA_VP5.0 and HEMA_AMPS5.0 gels. (**e**) Colorimetry results before and after cleaning using the studied gel formulations. The red line represents ∆E* limit for visual color-change perception. (**f**) Cleaning with HEMA_VP5.0 on white mock-up.

**Figure 5:**
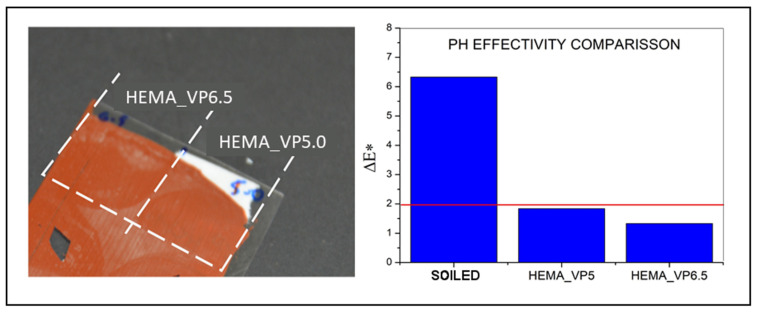
Cleaning test with HEMA_VP6.5 and HEMA_VP5.0 (left). Colorimetry results for soiled and cleaned surfaces with HEMA_VP5.0 and HEMA_VP6.5 gels.

SEM images were taken before and after cleaning of the soiled surfaces for comparison with the cleaned areas. The most representative images of the resulting surfaces after cleaning are shown in Figure 4a. Cleaning with HEMA_VP5, HEMA_VP6.5, HEMA_AMPS5 and HEMA_AMPS6.5 gels showed the best results regarding efficient soil removal, not wetting the surface and inducing no color change of the surface. As shown in Figure 4e, hydrogels with the best cleaning capability were also those that showed the best optical performance. According to UNE EN 15886:2011, the ∆E* value should be below 2 (red line in Figure 5) for the color variation to not be perceptible to the human eye.

### 4.4. Testing the Cleaning Efficiency According to pH

To check the effect of the pH on the cleaning efficiency [6], hydrogel formulations were cured by using two different pH-adjusted water solutions, with pHs at the upper and lower security range for acrylic emulsion paints (pH 5 and pH 6.5) [6,18]). According to previous works, gels with higher pH were expected to perform a deeper albeit more aggressive cleaning [27,29]. However, as shown in Figure 5, no significant differences were observed. The cleaning results for HEMA_VP gels are shown in Figure 5, which are consistent for all the formulations. Macroscopic results showed a similar cleaning efficiency for both pH values, supported by colorimetry data. Thus, pH 5.0 hydrogels were chosen since they achieved good cleaning results while maintaining the system in lower (safer) values of pH.

### 4.5. Agar Gels vs. UV-Cured Hydrogels

The cleaning performance of UV-cured hydrogels was compared with a reference agar hydrogel loaded with a pH 5.0 buffered aqueous solution. The agar hydrogel was prepared in a 3% aqueous buffer solution, as described elsewhere [47]. Agar gel is very popular among conservators since it is easy to prepare out of laboratory conditions, which is more attractive for users. However, the higher water absorption and the syneresis property [48] of agar gels led to a less controllable release of liquid that remains on the surface after removing the gel (see Figure 5) [40]. This carries several drawbacks for water-sensitive paints [49]. In contrast, UV-cured hydrogels have proven to be highly retentive, allowing the controlled action of the aqueous solutions even on highly sensitive substrates. The water release capacity of both UV-cured hydrogels and agar gels was measured gravimetrically, for comparison. Fully swollen gels were placed on a Petri dish over absorbent paper and covered with a mylar sheet to avoid water evaporation. After 30 min, water release was determined by weighing the filter paper before and after the application. The measurements were repeated three times.

The water release rate of the UV-cured hydrogel and of the agar gel is reported in Table 3. The excess wetting of the surface when using agar is evidenced by the water remaining on the surface together with the gravimetric results.

**Figure 6:**
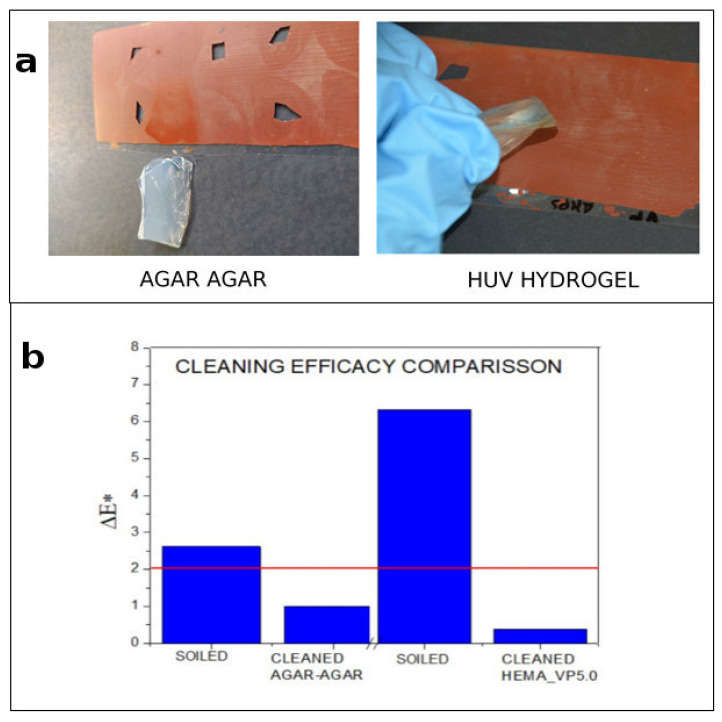
(**a**) Cleaning with ph 5.0 buffer confined in agar agar (left), cleaning with HEMA_VP5.0 (right). (**b**) Comparison of colorimetry results before and after cleaning with pH 5.0 agar-agar and UV-cured gels.

As shown in Figure 6, UV-cured hydrogels were able to achieve effective cleaning without wetting the surface. Colorimetry results showed that soiling removal when using UV-cured gels was even more efficient after surface water evaporation.

### 4.6. Gel Working Life/Number of Uses

**Figure 7:**
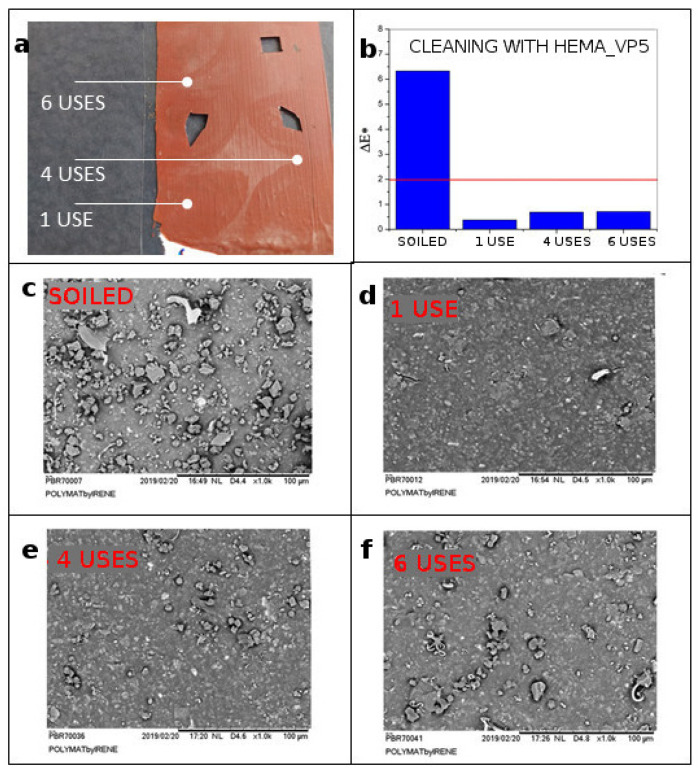
(**a**) Cleaning results after 1, 4 and 6 uses of the same HEMA_VP5.0 gel. (**b**) Colorimetry results of the cleaning after several uses. (**c**–**f**) SEM images of the surface before and after cleaning with gels that have been repeatedly used.

The number of effective applications using the same hydrogel has been explored so as to determine its working life. We selected the HEMA_VP5 hydrogel for this test, since it showed the best cleaning performance. The same side of the hydrogel was applied several times on the artificially soiled paint mock-up. As shown in Figure 7, as the number of uses increases, the dirt removal is less effective. However, even after six applications without a washing step between applications, the result remains acceptable, as can be observed on the colorimetry plot. Figure 7b shows how the hydrogel maintains its effectiveness after the sixth use, since ∆E* remains below two. This shows that the hydrogel is capable of removing dirt several times without needing rinsing. Obviously, the number of uses could vary depending on the type and amount of dirt present on the surface.

## 5. Discussion and Conclusions

In this article, we report a new family of easy-to-make UV-cured hydrogels for dry cleaning on water-sensitive paints. The hydrogels show good performance as efficient and safe cleaning tools for water-sensitive paint surfaces. The main advantage of the hydrogels presented here is that they are easy to make and they only need a UV lamp to prepare them. They could therefore trigger the incorporation of chemical gels in the daily practice of the art conservator–restorer. A wide range of formulations was tested, from which acrylic hydrogels based on HEMA-VP and HEMA-AMPS monomers were selected since they proved to be more adequate for the requirements posed by the conservation field. Their performance is similar to that of other chemical gels proposed before, with the advantage of not needing a complex laboratory process to create them.

Interestingly, UV hydrogels did not present wet residue problems after cleaning, and the pH of choice did not seem to affect the cleaning performance. In addition, water release is under control due to the good retaining properties of the hydrogel, which showed a reduction of moistening compared to other commonly used easy-to-make gels. Moreover, no extra aqueous solution needs to be loaded in order to achieve good cleaning efficiency. In conclusion, these hydrogels are very easy to make, are tunable, and they provide conservators with an optimal and comfortable cleaning system, thus contributing to the wide use of chemical hydrogels among conservators.

## Figures and Tables

**Table 1:** Weight percent composition of the UV-cured hydrogels; 2% weight based on monomer (wbm) of IRGACURE 2956 was added to each solution.

Name	Monomer 1 (wt %)	Monomer 2 (wt %)	Crosslinker (wt %)	Aqueous Solution (wt %)
HEMA_5	HEMA (49.75)	-	PEGDMA (0.25)	pH 5.0 buffer (50)
HEMA_VP5	HEMA (39.8)	VP (10)	PEGDMA (0.25)	pH 5.0 buffer (50)
HEMA_METAC 5	HEMA (39.8)	METAC (10)	PEGDMA (0.25)	pH 5.0 buffer (50)
HEMA_AMPS5	HEMA (39.8)	AMPS (10)	PEGDMA (0.25)	pH 5.0 buffer (50)
HEMA_6.5	HEMA (49.75)	-	PEGDMA (0.25)	pH 6.5 buffer (50)
HEMA_VP6.5	HEMA (39.8)	VP (10)	PEGDMA (0.25)	pH 6.5 buffer (50)
HEMA_METAC6.5	HEMA (39.8)	METAC (10)	PEGDMA (0.25)	pH 6.5 buffer (50)
HEMA_AMPS6.5	HEMA (39.8)	AMPS (10)	PEGDMA (0.25)	pH 6.5 buffer (50)

**Table 2:** Gravimetric results of water release from UV-cured hydrogels.

Name	gr/cm^2^
HEMA_METAC	0.03
HEMA_AMPS	0.02
HEMA_VP	0.026

**Table 3:** Gravimetric results of water release of UV-cured hydrogels and agar-agar gels.

gr/cm²	
HEMA_VP	0.006
AGAR	0.036
